# CaMKII inhibition reduces isoproterenol-induced ischemia and arrhythmias in hypertrophic mice

**DOI:** 10.18632/oncotarget.15099

**Published:** 2017-02-04

**Authors:** Ying Feng, Jun Cheng, Baozhu Wei, Yanggan Wang

**Affiliations:** ^1^ Department of Cardiology, Zhongnan Hospital of Wuhan University, Wuhan, China; ^2^ The Medical Research Institute of Wuhan University, Wuhan, China

**Keywords:** arrhythmia, CaMKII, cardiac hypertrophy, adrenergic stimulation, Pathology Section

## Abstract

**Objectives:**

The Ca/calmodulin-dependent protein kinase II (CaMKII), an arrhythmogenic molecule, is excessively activated in cardiac hypertrophy. Here, we investigated the effect of CaMKII inhibition in isoproterenol (ISO)-induced arrhythmias in hypertrophic mice.

**Results:**

ISO induced multiple types of arrhythmias in the hypertrophic mice but not in the normal mice. The QTc intervals were prolonged and the amplitudes of T waves were increased significantly by ISO prior to arrhythmia initiation. Inhibition of CaMKII prevented ISO-induced QTc prolongation and T wave elevation and abrogated arrhythmia induction.

**Materials and Methods:**

Pressure-overload cardiac hypertrophy was induced in mice by thoracic aortic banding. Arrhythmias were recorded by electrocardiogram in conscious mice.

**Conclusions:**

CaMKII inhibition is effective in suppressing adrenergic activation-induced ventricular arrhythmias in cardiac hypertrophy, of which the ventricular ischemia-induced CaMKII activation plays an important role.

## INTRODUCTION

Heart failure (HF) is featured with high mortality and morbidity and healthcare expenditures due to the ineffective therapy. Half of HF patients died of sudden cardiac death (SCD), of which ventricular arrhythmias are the most common cause [1]. HF is often a transition from cardiac hypertrophy, a process initiated as compensation to pressure-overload. In the setting of left ventricular hypertrophy (LVH), the incidence of ventricular arrhythmias is significantly increased [2]. Thus, LVH is regarded as an independent risk factor of SCD [3].

Recent studies in animal models revealed that Ca^2+^/calmodulin dependent protein kinase II (CaMKII) is excessively activated in cardiac hypertrophy and that CaMKII overexpression can induce cardiac hypertrophy, progressive HF and lethal ventricular arrhythmias [4]. In contrast, inhibition of CaMKII protects against structural heart disease and inhibits ventricular arrhythmias [5]. In addition, persistent activation of β-adrenergic pathway, a known case in cardiac hypertrophy and failure, can activate CaMKII and enhance Ca^2+^ cycling, which may trigger ventricular arrhythmias. In the present study, we developed a pressure-overload LVH mouse model and utilized the conscious electrocardiogram (ECG) to monitor the occurrence of ventricular arrhythmias in mice under ISO stimulation. We also investigated the effects of CaMKII inhibition on suppression of the ventricular arrhythmias in LVH mice and explored the possible underlying mechanisms.

## RESULTS

### Cardiac hypertrophy development

Two weeks after TAB, cardiac hypertrophy has been developed in mice and confirmed by echocardiography measurements. The morphometric data from each group was illustrated in Table 1. Compared with sham group, the TAB group, TAB+KN93 group and TAB+KN92 group showed thicker LV anterior and posterior wall, larger LV mass and heart weight to body weight ratio (HW/BW ratio) without changes in the systolic function.

**Table 1:** Baseline echocardiography parameters and HW/BW ratio

Parameter	Sham (*n*= 10)	TAB (*n*= 10)	TAB+KN93 (*n*= 10)	TAB+KN92 (*n*= 10)
LVAW; s (mm)	1.2762 ± 0.042	2.0343 ± 0.095*	2.1260 ± 0.187*	2.0912±0.136*
LVAW; d (mm)	0.6953 ± 0.049	1.4345 ± 0.107*	1.4633 ± 0.186*	1.4562±0.164*
LVPW; s (mm)	1.1893 ± 0.086	2.1335 ± 0.164*	2.0915 ± 0.115*	2.1134±0.209*
LVPW; d (mm)	0.8648 ± 0.045	1.5865 ± 0.114*	1.5583 ± 0.087*	1.5673±0.102*
LV mass (mg)	67.2450 ± 1.778	100.6368 ± 2.380*	98.0320 ± 2.246*	101.3857±3.234*
H/BW ratio (mg/g)	5.1563 ± 0.093	7.3020 ± 0.301*	7.4289 ± 0.197*	7.3576±0.298*
EF (%)	93.5 ± 2.3	92.7 ± 2.9	93.7 ± 1.4	93.6±1.8
FS (%)	67.6 ± 2.2	62.3 ± 5.7	66.7 ± 3.3	65.9±4.5

* *P* < 0.05, compared with sham group

Abbreviations: LVAW: left ventricular anterior wall; LVPW: left ventricular posterior wall; EF: ejection fraction; FS: fractional shortening; s: systolic; d: diastolic.

### ISO-induced arrhythmias in hypertrophic mice

**Figure 1:**
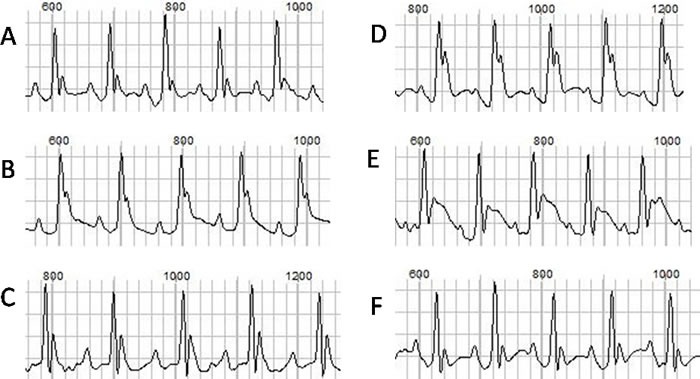
The ECGs at baseline and after drug injection **A**., **B**. and **C**. represent the baseline ECGs from the normal, hypertrophic and hypertrophic mouse after KN93 injection, respectively. **D**., **E**. and **F**. represent the ECGs after ISO injection, respectively.

**Figure 2:**
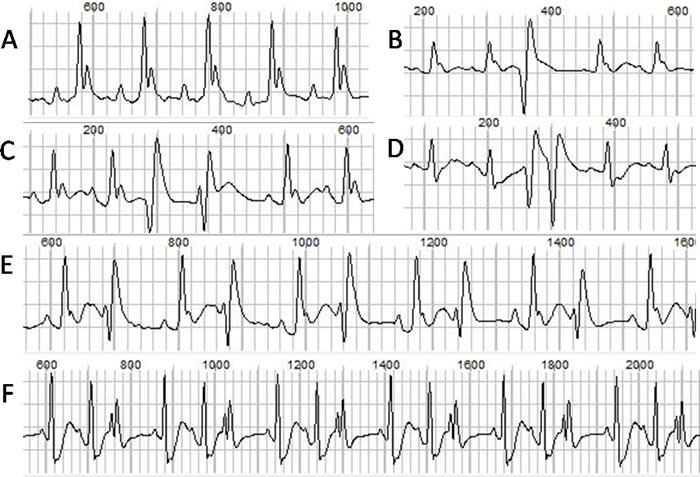
The normal ECG and different types of ventricular arrhythmias **A**. normal ECG. **B**. single PVC. **C**. heteromorphic paired PVCs. **D**. paired PVCs. **E**. bigeminy. **F**. trigeminy.

The ECGs at baseline and after ISO injection from each group were recorded and analyzed. The representative ECGs were showed in Figure 1. Ventricular arrhythmias were readily induced by ISO stimulation in TAB group, but not in the sham group. The typical arrhythmias were showed in Figure 2. Before ISO stimulation, the arrhythmia scores for the sham group and TAB group were 0.67 ± 0.52 and 1.67± 0.52, respectively (*P* > 0.05). After ISO stimulation, the scores were 1.33 ± 0.82 and 6.17 ± 1.50, respectively (*P* < 0.05).

### CaMKII inhibition suppresses ISO-induced arrhythmias in hypertrophic mice

To understand the role of CaMKII activation in the ISO-dependent arrhythmias, we tested the effect of CaMKII inhibition by KN 93. Meanwhile, we used KN92, an inactive analogue of KN93, as a negative control to rule out the off-target effect of KN93. We found that application of KN93 (10 μmol/kg IP, 10 min prior to ISO injection) significantly suppressed ISO-induced ventricular arrhythmias in the hypertrophic mice, while application of KN92 had no effect. Before ISO stimulation, there was no significant difference in the arrhythmia scores among the TAB group, TAB+KN92 group and TAB+KN93 group (1.60 ± 0.52, 1.10±0.99, and 1.20 ± 0.42, respectively; *P* > 0.05). After ISO stimulation, however, arrhythmia was increased in TAB and TAB+KN92 mice (the scores were 6.00 ±1.50 and 5.10±1.60, respectively) but inhibited in TAB+KN93 mice (the score was 2.90 ± 0.74; *P* < 0.05 compared with TAB and TAB+KN92 mice).

### CaMKII inhibition reduces ISO-induced arrhythmias by reducing cardiac response to ischemia

**Figure 3:**
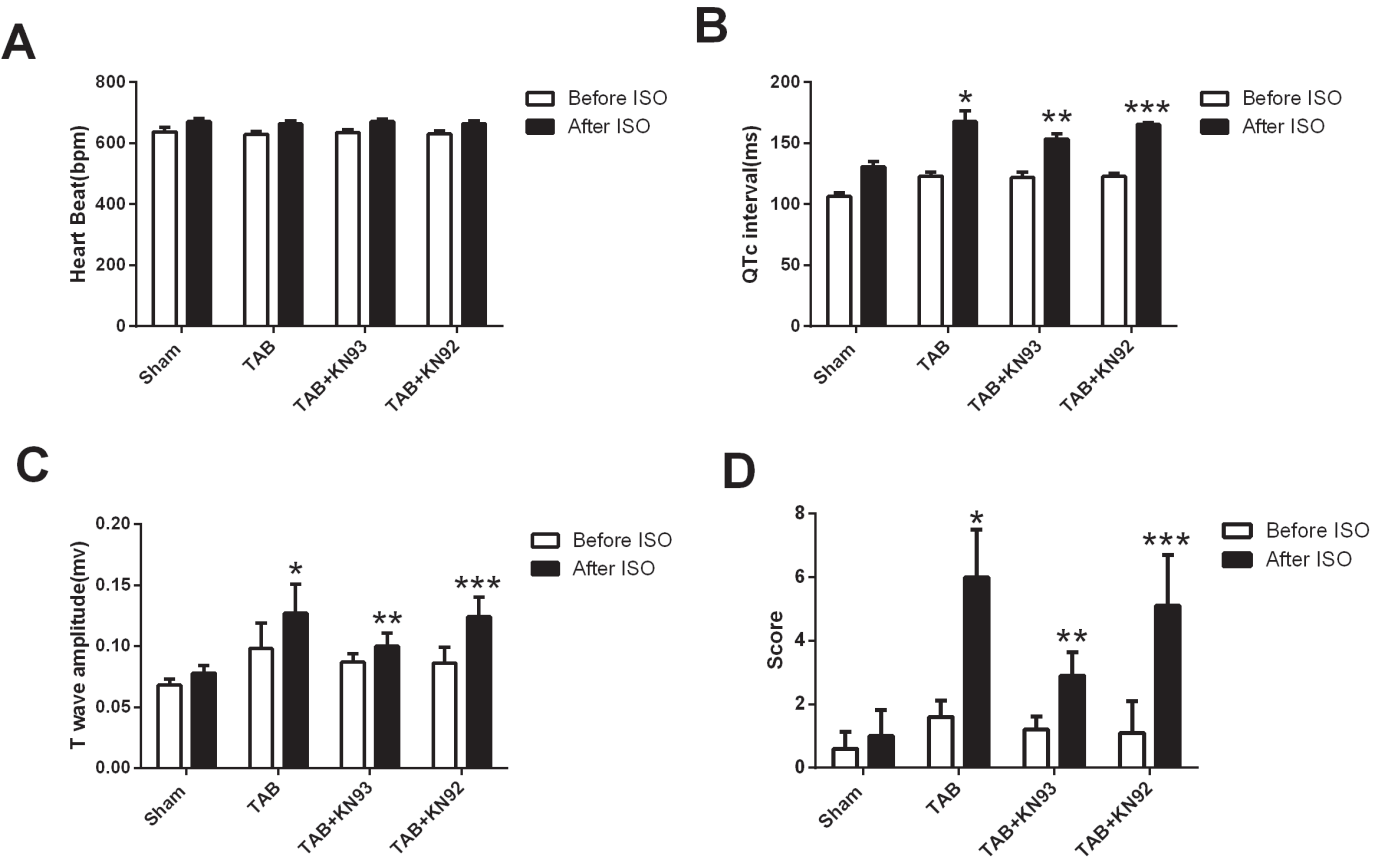
The bar graph illustrating the changes in heart rate, QTc interval, T wave amplitude and arrhythmia scores *: *P* < 0.05, compared with the sham mice. **: *P* < 0.05, compared with TAB mice. ***: *P* < 0.05, compared with the TAB+KN93 mice.

In order to understand the mechanism underlying ISO-induced arrhythmias in hypertrophic mice, we analyzed the ISO-induced changes in heart rate (HR), QTc interval and T wave amplitude. We found that ISO induced a significant increase in the HR and these changes were similar in all of the 4 groups (Figure 3A), suggesting that increasing of HR is unlikely responsible for ISO-induced arrhythmias. These results also suggest that cardiac hypertrophy and CaMKII activation do not alter the adrenergic regulation of HR. However, we found that the QTc interval was prolonged exclusively in the hypertrophic mice, and that KN93 treatment reduced the ISO-induced QTc further prolongation (Figure 3B). Furthermore, T wave was dramatically elevated in the hypertrophic mice (Figure 3C), and ISO caused a much greater increase in T wave amplitude in the hypertrophic mice than in the normal mice, and this increase was attenuated by KN93 but not by KN92 treatment. The changes in QTc interval and T wave amplitude were consistent with the arrhythmia scores (Figure 3D).

## DISCUSSION

Ventricular arrhythmia is a common cause of SCD in patients with structural heart disease, especially under cardiac stress [6,7]. In the hypertrophic heart, the cardiac ischemia is persistent due to the enlarged myocytes and the increased distance to cardiac capillaries. Meanwhile, it is known that in cardiac hypertrophy, the sympathetic tone is increased with excessive activation of CaMKII [8]. Although the hypertrophic heart is known susceptible to arrhythmia induction, the underlying mechanisms are poorly understood. Our study showed that ISO could steadily induce ventricular arrhythmias in the hypertrophic mice with a significant increase in the QTc interval and T wave amplitude. These results indicate that ISO-induced ventricular arrhythmias in hypertrophic mice are likely associated with the ISO-induced cardiac ischemia. Indeed, CaMKII inhibition effectively suppressed the ISO-induced ventricular arrhythmias in hypertrophic mice by attenuation of ISO-induced further increase in QTc interval and T wave amplitude. Importantly, the changes in QTc interval and T wave well corroborate to the arrhythmia scores.

Cardiac ischemia in mice can be assessed using conscious surface ECG by analyzing the QTc interval and T wave amplitude [9]. Scofield et al. demonstrated that after left anterior descending (LAD) coronary artery occlusion, QTc intervals were widened and T waves were elevated in mice using surface pad ECG [10]. In this study, we observed similar changes in QTc interval and T wave by ISO administration, indicating the involvement of myocardial ischemia herein.

CaMKII plays an important role in pressure overload-induced hypertrophy by transcriptional regulation of hypertrophic genes [11]. Meanwhile, CaMKII inhibition prevents arrhythmias *in vivo* in cardiac hypertrophy in the spontaneously hypertensive rats [12] and in the transgenic mice overexpressing CaMKIIδc [13]. A recent study reported that CaMKII activation promotes ischemia/reperfusion (I/R) injury [14]. However, it is unknown whether CaMKII-mediated arrhythmias in cardiac hypertrophy are associated with cardiac ischemia. For the first time, we demonstrated that acute inhibition of CaMKII attenuates ventricular arrhythmias induced by adrenergic stimulation in cardiac hypertrophy by suppressing ISO-induced myocardial ischemia.

It is known that CaMKII activation induces arrhythmias in structural disease by activation of multiple substrates, including modulation of several ion channels and transporters [15]. Our results here point out a novel mechanism underlying CaMKII mediated ventricular arrhythmias in structural heart disease under sympathetic stress. However, the actual mechanism how CaMKII inhibition reduces ischemic response to adrenergic stimulation in diseased heart remains unclear, and this could be a new direction for further studies.

## MATERIALS AND METHODS

### Thoracic aortic banding

Mice were housed under standard conditions and all experimental procedures were approved by the local Institutional Committee for Care and Use of Laboratory Animals (IACUC) at Zhongnan Hospital of Wuhan University, Wuhan. Mice were bought from Animal experiment center of Wuhan University. We used thoracic aortic banding (TAB) [16] to establish the pressure-overload cardiac hypertrophy mouse model with preserved heart function according to the protocols approved by the institution's Animal Care and Use Committee of Wuhan University. In brief, male C57B/L6 mice (6 weeks old) were anesthetized with phenobarbital sodium (60mg/Kg, IP). A 3mm thoracotomy was created at the left second intercostal space. The transverse aortic arch was ligated between the innominate and left common carotid arteries with an overlying 27-Gauge needle using 7-0 Prolene, and the needle was then removed, leaving a discrete region of stenosis. Then the chest was closed, leaving two weeks’ time to develop cardiac hypertrophy. The control group went through sham operation. All animals received humane care.

### Echocardiography

The mice were trained daily for 7 days with practice handling and echocardiograph (Visualsonics, Vevo 2100, Canada) imaging before data collection in order to minimize the stress-induced artifact. Prior to acquisition of echo images, the chest hair was removed by hair removal cream. The echo transmission gel was pre-warmed, and the operation was gentle to avoid the reflex induced by the transducer pressure on the chest. Mice were imaged in the supine position, and the M-mode images were taken from the short axis at the level of the largest LV diameter. The following parameters were obtained from the M-mode tracings, including LV anterior and posterior wall thickness at diastole and systole (LVAW;d, LVAW;s, LVPW;d, LVPW;s), LV mass, ejection fraction (EF) and fractional shortening (FS).

### Arrhythmia induction and CaMKII inhibition

Awake mice were placed in the ECG recording unit (Emka Technology, France) to record baseline ECG for 30min. The sham group and TAB group were given an intraperitoneal injection (IP) of ISO (3 mg/kg body weight). The ECG was recorded continuously for 1h and analyzed offline. In another set of experiments, mice in the TAB+KN93 group were first injected with CaMKII inhibitor KN93 (10 μmol/kg body weight), while mice in the TAB+KN92 group were first injected with the same dose of KN92 (a KN93 inactive analogue) and followed by a subsequent injection with ISO 10 min later. The conscious ECG was continuously recorded for 1h for arrhythmia analysis.

### Statistics

To evaluate the arrhythmic susceptibility and severity, arrhythmias were classified into several types with scores: 1) single ventricular premature contraction (PVC), 1 score; 2) paired PVC, 2 scores; 3) bigeminy or trigeminy or paroxysmal ventricular tachycardia (> 3 continuous PVCs), 3 scores; 4) continuous ventricular tachycardia (> 10 continuous PVCs) or polymorphic ventricular tachycardia, 4 scores. Data are presented as mean± SD. Student-*t* test was performed in paired analyses, ANOVA was performed between groups, and *P* <0.05 was considered statistically significant.
